# Cytotoxic KLRG1+ IL-7R- effector CD8+ T cells distinguish kidney transplant recipients controlling cytomegalovirus reactivation

**DOI:** 10.3389/fimmu.2025.1542531

**Published:** 2025-02-14

**Authors:** Yumeng Sun, Subha Sen, Rajesh Parmar, Janice Arakawa-Hoyt, Monica Cappelletti, Maura Rossetti, David W. Gjertson, Tara K. Sigdel, Minnie M. Sarwal, Joanna M. Schaenman, Suphamai Bunnapradist, Lewis L. Lanier, Harry Pickering, Elaine F. Reed

**Affiliations:** ^1^ Department of Pathology and Laboratory Medicine, University of California, Los Angeles, Los Angeles, CA, United States; ^2^ Department of Microbiology and Immunology, Parker Institute for Cancer Immunotherapy, University of California, San Francisco, San Francisco, CA, United States; ^3^ Department of Surgery, Division of Multi Organ Transplantation, University of California, San Francisco, San Francisco, CA, United States; ^4^ Division of Infectious Diseases, David Geffen School of Medicine, University of California, Los Angeles, Los Angeles, CA, United States; ^5^ Division of Nephrology, David Geffen School of Medicine, University of California, Los Angeles, Los Angeles, CA, United States

**Keywords:** kidney transplantation, cytomegalovirus (CMV), single-cell RNA sequencing (sc-RNA seq), CD8+ T cells, effector T cell differentiation

## Abstract

**Introduction:**

Cytomegalovirus (CMV) viremia remains a major contributor to clinical complications in solid organ transplant (SOT) patients, including organ injury, morbidity and mortality. Given their critical role in antiviral defense, CD8+ T cells are essential for protective immunity against CMV.

**Methods:**

Using single-cell RNA sequencing, we investigated the transcriptional signatures and developmental lineages of CD8+ T cells in eight immunosuppressed kidney transplant recipients (KTRs) who received organs from CMV-seropositive donors. Results were validated in a cohort of 62 KTRs using immunophenotyping.

**Results:**

Our data revealed a significant influence of CMV serostatus on transcriptional variance of CD8+ memory T cells, associating with the first principal component from a global analysis of CD8+ T cells (p =0.0406), forming a continuum with five principal differentiation trajectories driven by CMV primary infection or reactivation. Following CMV primary infection, CD8+ T cells were hallmarked by restrained effector-memory differentiation. CD8+ T cells during CMV reactivation diverged non-linearly into senescent-like cells with signatures of arrested cell cycle, diminished translational activity and downregulated *ZNF683* and longitudinally expanding effector cells with robust cytotoxic potential and upregulated *ZNF683*, acting as a reservoir for long-lived effector cells supporting long-term protection. Notably, CD28^lo^ KLRG1^hi^ IL-7R (CD127)^lo^ HLA-DR^hi^ CD8+ T cells present prior to the detection of viremia in CMV-seropositive patients emerged as a key feature distinguishing patients who did or did not undergo CMV reactivation after prophylaxis discontinuation (p =0.0163). Frequencies of these cells were also positively correlated with CMV-stimulated secretion of IFN-γ (p =0.0494), TNF-α (p =0.0358), MIP-1α (p =0.0262), MIP-1β (p =0.0043).

**Discussion:**

These results provide insights into the transcriptional regulation that influences the generation of CD8+ T cell immunity to CMV and may inform strategics for monitoring host immune response to CMV to better identify and introduce therapeutic intervention to patients at risk of developing clinically significant CMV viremia.

## 1 Introduction

Cytomegalovirus (CMV) infection is a globally prevalent viral pathogen and causes mostly quiescent infections in immunocompetent individuals. However, in immunocompromised solid organ transplant (SOT) and hematopoietic stem cell transplant (HSCT) recipients CMV can cause severe organ-invasive disease, morbidity, and mortality (1, 2). The risk of experiencing post-transplant (Tx) CMV infection is stratified by pre-transplant CMV serostatus of organ donors and recipients, defined by the presence of circulating anti-CMV antibodies. CMV-seronegative transplant recipients (R-) have the highest risk of CMV infection if they receive organs from CMV-seropositive donors (D+) and have the worst death-censored patient and graft survival rates; CMV-seropositive recipients (R+) carry the latent virus and bear the second highest risk for CMV transmission and reactivation post-Tx (3–5). CMV causes remarkable changes in host immunity, including sustained CD8+ T cell expansion, a phenomenon termed memory inflation, that plays a critical role in controlling CMV Infection (6, 7).

Although immunophenotypes of circulating CD8+ T cells following antigen stimulation *in vitro* have been subdivided into subsets by their expression of canonical markers and their degree of differentiation increasing from naïve T cells to stem-like memory T cells (TSCM), central memory T cells (CM), effector memory T cells (EM), and terminal effector memory T cells (TEMRA), the identification of heterogeneous mixtures of rare or transitional T cell subsets have led to an alternative concept that T cell phenotypes exist in a continuum that is shaped by the type of antigen, infection history, and by systemic inflammation elicited during antigen encounter (8–12). The effector-memory phenotypes induced by CMV comprise a highly heterogenous population of T cells, and the extent to which these cells provide a protective advantage remains poorly understood. While commonly used antiviral drugs and prophylactic regimens are effective in preventing and treating CMV disease, they may cause significant side effects and drug resistance and demand additional management in case of refractory CMV infection (13, 14). Further investigation into incorporating cellular immune parameters into clinical risk stratification could enhance CMV management and improve patient outcomes.

In this study, we assessed the spectrum of phenotypic and transcriptomic states that circulating CD8+ T cells assume and their associated functions during CMV infection in kidney transplant recipients (KTRs). We analyzed the temporal phenotypic states of CD8+ T cells in a cohort of D+R+ KTRs experiencing CMV reactivation (R+, PCR+) and compared them to CD8+ T cells from D+R- KTRs experiencing primary infection (R-, PCR+). Our data identified a restrained CD8+ T cell effector-memory differentiation state during primary infection. During CMV reactivation, CD8+ T cells expanded senescent-like cells and robust, cytotoxic effector cells with the latter showing CD8+ CD28^lo^ KLRG1^hi^ CD127^lo^ HLA-DR^hi^ phenotypes that distinguished PCR+ and PCR- patients before detection of CMV viremia. Our data provide deep insight into the molecular characteristics of CD8+ T cells emerging from CMV viral infection and may guide definition and monitoring of host immunological response to CMV to better predict patients who will develop clinically significant CMV viremia.

## 2 Materials and methods

Additional materials and methods may be found in **Supplementary Materials and Methods**.

### 2.1 Study population and design

The study design included IRB consented KTRs with (PCR+) and without CMV viremia (PCR-) enrolled 2013-2015 in a single center retrospective UCLA cohort of 62 KTRs (IRB#11-001387), including 31 CMV PCR+ KTRs and propensity-score matched CMV PCR- KTRs. CMV PCR+ was defined as the presence of CMV DNA exceeding 137 IU/mL in the patient’s blood through PCR test (Cobas AmpliPrep/Cobas TaqMan CMV test, Roche). Patients received either anti-thymocyte globulin (ATG) or basiliximab for induction therapy and were maintained on triple immunosuppression, including tacrolimus, mycophenolate mofetil, and prednisone. All KTRs were sampled 3 and 12 months after transplant, with additional samples 1 week and 1 month after CMV viremia for PCR+ individuals. Patient clinical characteristics are summarized in **Table 1** and previously described (15).

**Table 1:** Patient demographics.

	CMV PCR+ (n=31)	CMV PCR- (n=31)	Sc-RNAseq^B^ CMV PCR+ (n=4)	Sc-RNAseq^B^ CMV PCR- (n=4)
Recipient mean age (range)	54.4 (22-77)	54.4 (30-74)	57.0 (22-59)	56.5 (30-74)
Recipient female (%)	10 (32.3%)	12 (38.7%)	2 (50%)	2 (50%)
Race/ethnicity Asian Black or African American Hispanic or Latino Other^A^ White	6 (19.4%) 7 (22.6%) 9 (29.0%) 2 (6.5%) 7 (22.6%)	4 (12.9%) 3 (9.7%) 9 (29.0%) 3 (9.7%) 12 (38.7%)	1 (25%) 1 (25%) 0 (0%) 1 (25%) 1 (25%)	0 (0%) 0 (0%) 0 (0%) 1 (25%) 3 (75%)
Received ATG Yes No	9 (29.0%) 22 (71.0%)	9 (29.0%) 22 (71.0%)	0 (0%) 4 (100%)	0 (0%) 4 (100%)
Donor type Deceased Living	17 (54.8%) 14 (45.2%)	15 (48.4%) 16 (51.6%)	2 (50%) 2 (50%)	0 (0%) 4 (100%)
Rejection in 1 year No Yes	26 (83.9%) 5 (16.1%)	27 (87.1%) 4 (12.9%)	3 (75%) 1 (25%)^C^	4 (100%) 0 (0%)
Recipient CMV Serostatus Seropositive (R+) Seronegative (R-)	24 (77.4%) 7 (22.6%)	24 (77.4%) 7 (22.6%)	2 (50.0%) 2 (50.0%)	2 (50.0%) 2 (50.0%)
Median days after transplant (IQR) Baseline Long-term 1-week post-viremia 1-month post-viremia	55 (39-78) 345 (287-413) 92 (56-167) 176 (88-277)	90 (85-159) 363 (321-381) - -	62 (50-73) 359 (300-375) 125 (81-167) -	153 (118-186) 309 (285-332) - -
GFR decline from 6 to 12-month (median, IQR)	8.4% (3.2%-11.5%)	9.8% (3.1%-23.5%)	12.1 (4.6%-22.8%)	20.8% (9.8%-32.5%)

^A^Multiracial, Native Hawaiian, or Pacific Islander. ^B^Single-cell RNAseq was conducted on 8 of the 62 patients in the cohort. ^C^Acute rejection was diagnosed at the time of transplant and was given standard of care treatment. No rejection at the time of or after sampling.

### 2.2 Single-cell transcriptomic sequencing and analyses

CD8+ T cells were isolated from longitudinal PBMCs of eight KTRs (CD8+ T cell isolation kit, Miltenyi Biotec) and underwent single-cell RNA-sequencing (10X Genomics). Data were QC-ed and analyzed in R (version 4.3.2) using Seurat package (Version 4.3.0 for QC, batch-correction, normalization, scaling, dimension reduction and clustering, and Version 5.0.1 for differential expression analyses). Pseudotime analyses were performed using Monocle3 package (Version 1.3.1) (16–18).

### 2.3 Multi-color flow cytometry immunophenotyping

Cryopreserved PBMCs were thawed in pre-warmed RPMI 1640 (Gibco, 12633012) supplemented with 10% fetal bovine serum (FBS, Omega Scientific, FB-02), 1% penicillin-streptomycin (Gibco, 15140163), and washed with phosphate-buffered saline (PBS, Corning, 21040CM). Dead cells were labeled using viability dye (LIVE/DEAD Fixable Blue Dead Cell Stain Kit, Invitrogen L34962). PBMCs were then washed with FACS buffer (PBS with 1% heat-inactivated FBS) and pre-incubated with human TrueStain FcX (BioLegend, 422302) at 4°C for 30 minutes. Cells were subsequently stained with fluorochrome-conjugated antibodies (**Supplementary Table S1**) at 4°C for 30 minutes and fixed with Fluorofix buffer (BioLegend) following manufacturer’s procedures. Samples were acquired on a LSRFortessa™ Cell Analyzer (BD Bioscience) and raw FCS files were imported into R and analyzed as described below, using R packages ConsensusClusterPlus (19), flowCore (20) and flowWorkspace (20). Dead cells and doublets were removed, and raw mean fluorescence intensity (MFI) values were *arcsinh* transformed with a cofactor parameter of 150. Live CD3+CD8+ T cell subsets were identified in an unsupervised manner using the FlowSOM (21) algorithm, which initially defined 100 clusters using a Self-Organizing Map (SOM). These clusters were combined into 40 meta-clusters by hierarchical clustering.

### 2.4 CMV-stimulated cytokine and chemokine profiling

PBMCs were rested overnight and stimulated for 8 hours with anti-CD28 and anti-CD49a monoclonal antibodies (BD Biosciences, 347690), GolgiPlugTM Protein Transport Inhibitor (BD Biosciences, 555029) and one of the following stimuli: 1) no stimulation, 2) overlapping 15 amino acid peptide pools representing CMV virus proteins from the 9 most immunodominant antigens, including UL55, UL83 (pp65), UL99, UL36, UL48_sub1, UL48_sub2, UL122 (IE-1), UL123 (IE-2), and US32 (JPT Peptide Technologies, PM-Pan-CMVselect-1) at a final concentration of 5 μg/mL. Cell supernatants were collected and assessed for cytokines and chemokines via 38-plex Luminex multibead arrays (Millipore). To correct for background analyte production, finalized concentrations (pg/mL) of CMV-stimulated analytes were defined as the CMV-stimulated condition minus the unstimulated condition.

### 2.5 Statistical analyses

Longitudinal change in pseudotime in single-cell analysis with respect to time of sample collection was determined using mixed-effect linear regression, with patient ID as a random effect variable. Pseudotime change with respect to time and serostatus was determined by an interaction term of time and serostatus in the linear mixed-effect model. Longitudinal changes in flow cytometry-identified cell cluster and concentrations of CMV-stimulated cytokine with respect to time in PCR+ patients were determined by mixed-effect linear regressions with patient ID as a random effect variable. P-values comparing PCR+ and PCR- groups at one timepoint were determined by Mann-Whitney test. Correlation of CD28^lo^ KLRG1^hi^ CD127^lo^ HLA-DR^hi^ cluster frequency with CMV-stimulated cytokine production for each patient was determined by Spearman’s rank correlation coefficient. Results of statistical tests are indicated as p >0.05: ns; p <0.05: *; p <0.01: **; p <0.001: ***; p <0.0001: ****.

## 3 Results

### 3.1 Single-cell transcriptomic profiles of CD8+ T cells separated CMV primary infection and reactivation

**Figure 1:**
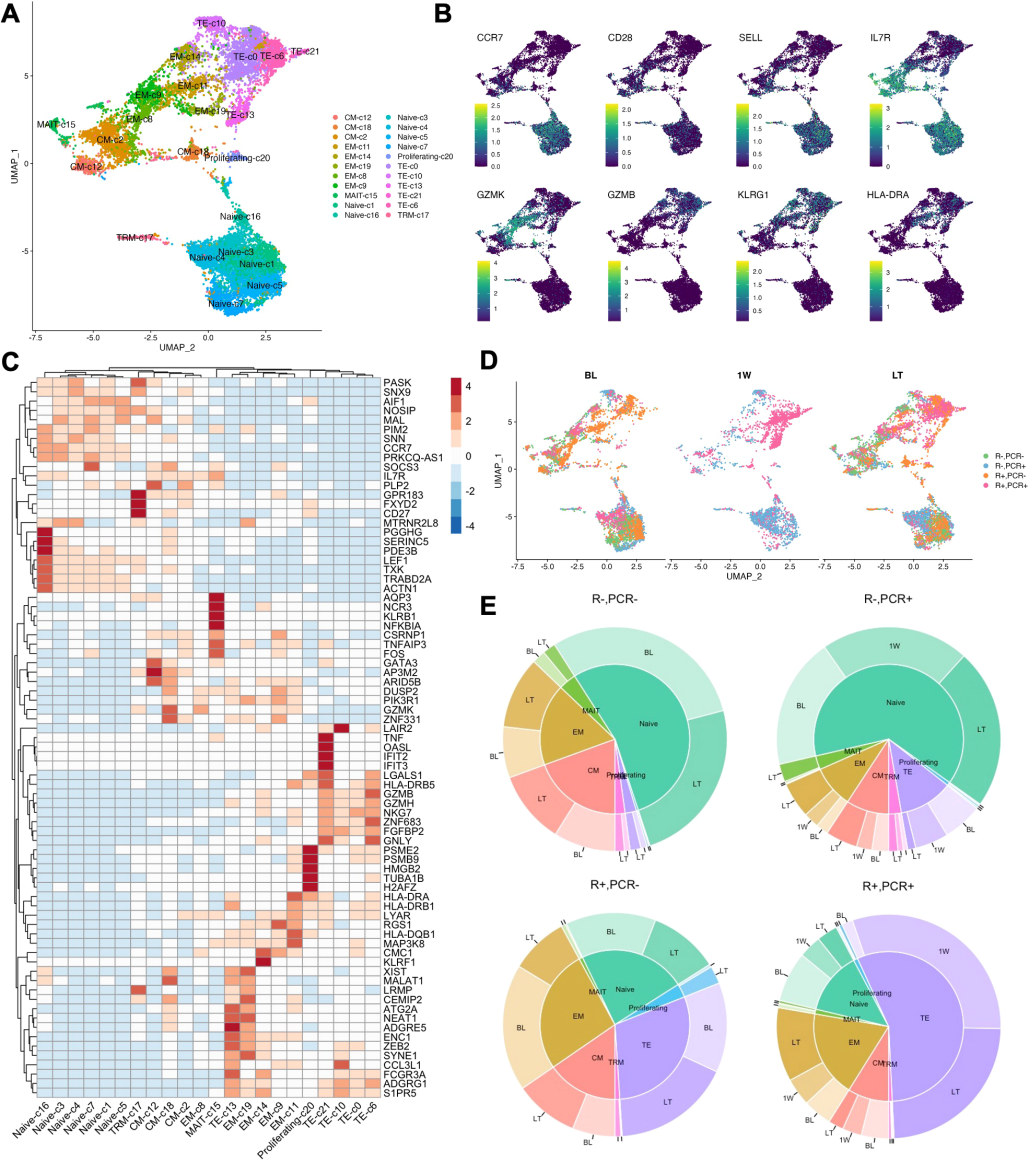
Single-cell RNA transcriptional landscape of CD8+ T cells in KTRs. **(A)** Single-cell RNA-sequencing (10X Genomics) was performed on isolated CD8+ T cells in a subset of CMV PCR+ (R-: n=2; R+: n=2) and matched PCR- control KTRs (R-: n=2; R+: n=2). QC-ed and annotated CD8+ T cell clusters were projected on UMAP. Colors encode cell cluster assignment. **(B)** Expression level of selected genes projected on UMAP. Colors encode low to high expression. **(C)** Heatmap of top5 DEGs (p <0.05, average Log2(Fold Change) >0.5) of each cluster. **(D)** UMAP of single-cell data separated by timepoint of collection. Colors encode patient CMV serostatus and viremia result. **(E)** Proportions of cells in cell subsets (pie chart) and at different timepoints (donut chart) from each group of patients.

To comprehensively characterize the longitudinal phenotypic changes in CD8+ T cells in KTRs experiencing CMV primary infection or reactivation, we performed single-cell RNA sequencing (scRNA-seq) on purified peripheral blood CD8+ T cells collected at pre-viremia baseline (BL, approximately 3-months post-Tx), 1-week post-viremia (1W), and long-term (LT, approximately 1-year post-Tx) from a selected cohort of R- (n=4) and R+ (n=4) KTRs (**Table 1**). After batch-correction and QC, all non-CD8+ T cells were removed and CD3+CD8+ cells were partitioned into twenty-two clusters and visualized by Uniform Manifold Approximation Projection (UMAP) (**Figure 1A**). We annotated these clusters based on differentially expressed gene markers and canonical gene expressions, including naïve (*CCR7*+ *CD28*+ c1, 3, 4, 5, 7, 16), proliferating (*MKI67*+, c20), central memory (CM, *CCR7*+ *SELL* (CD62L)+ *JUNB*+ *STAT3*+ c2, 12, 18), effector memory (EM, *CCR7- KLRG1+ GZMK+* c8, 9, 11, 14, 19), terminal effector (TE, *GZMB+ KLRG1+*, c0, 6, 10, 13, 21), tissue-resident memory (TRM, *ITGAE* (CD103)+, c17), and mucosal-associated invariant T (MAIT, *KLRB1*+ *CCR6*+, c15) cells (**Figures 1A, C**, **Supplementary Figure S1**). A pattern of increasing differentiation was seen from the lower region to the upper region on the UMAP (**Figure 1B**). Cells were distinctly separated by their CMV serostatus and PCR status: late-differentiated cells were enriched at BL and LT in both PCR- and PCR+ R+ patients, and at 1W R- PCR+ and R+ PCR+ patients were well-separated into distinct clusters (**Figure 1D**). Comparing proportions of different subsets of CD8+ T cells in each group of patients, we found CD8+ T cells from R+ patients had much larger proportions of EM and TE cells over other CD8+ T cell subsets, with R+ PCR+ patients having the most pronounced TE expansion (**Figure 1E**).

### 3.2 Pseudotemporal analysis defines multiple differentiation lineages and their associated transcriptomic profiles of CD8+ T cells

**Figure 2:**
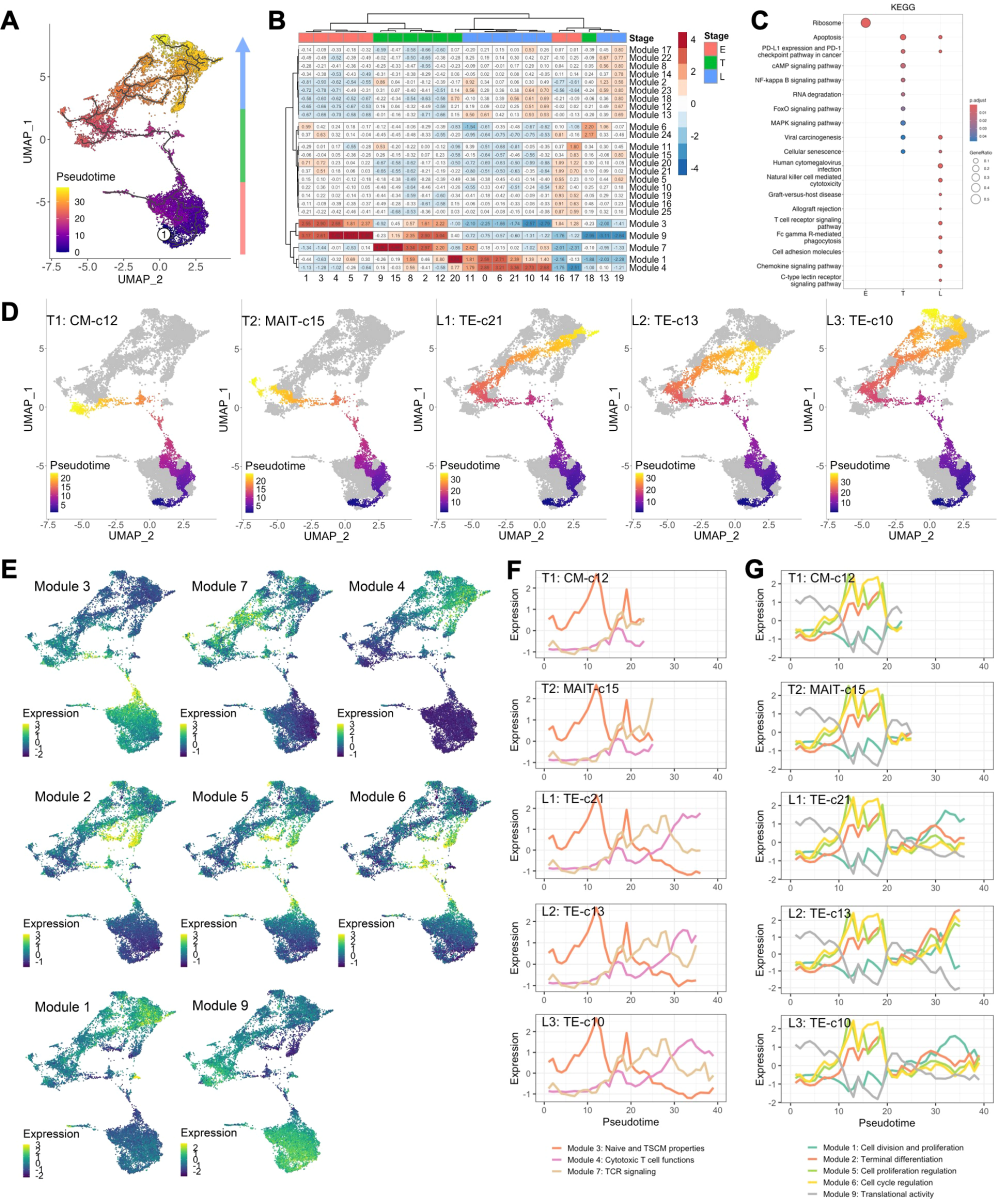
CD8+ T cell transcriptomes dictated by pseudotime. **(A)** Pseudotemporal trajectories (black lines) were constructed via Monocle 3 revealing the amount of transcriptional change that a cell undergoes from the starting state to the end state. Root node (①) indicates the start of the trajectories and was programmatically determined at the node with highest occupancy of cells from BL samples. Colors encode pseudotime, the path between a cell on trajectory and the root node. **(B)** Heatmap of gene module expressions of each cell cluster. Expression of differentially expressed genes (DEGs, q-value <0.05) across UMAP space were identified using spatial autocorrelation analysis were grouped into gene module via Louvian community analysis. Aggregated gene module expressions of each cluster were subject to clustering (Ward.D) into E-, T- and L-staged cells. **(C)** Enriched KEGG pathways using DEGs comparing E, T, and L-staged cells. **(D)** Lineage of cell differentiation defined by the track from root node to the ending nodes of clusters 12 (T1: CM-c12), 15 (T2: MAIT-c15), 21 (L1: TE-c21), 13 and 19 (L2: TE-c13), and 10 and 14 (L3: TE-c10). **(E)** Aggregated expression of gene modules of individual cells projected on UMAP. **(F, G)** Expression of aggregated modules with respect to pseudotime of cells in lineage T1, T2, L1, L2 and L3. Lines indicate the centroid module expressions of cells within pseudotime window of 1.

Though regions of variable CD8+ T cell differentiation can be broadly defined, cells distributed based on continuous expression of their canonical T cell markers and partitioned into more distinct and heterogeneous clusters of TE phenotype (**Figure 1A**). To define progression along the transcriptional continuum of CD8+ T cell differentiation, we placed the single cells on pseudotime trajectory [Monocle 3 (16–18)] originating from root node ①, the computationally-defined region of least differentiated cells (**Figure 2A**, **Supplementary Figure S2A**). We used spatial autocorrelation analysis to identify differentially expressed genes (DEGs) along the trajectories and their associated gene modules (**Figure 2B**, **Supplementary Table S2**). By further clustering the 22 defined cell clusters based on aggregated module expression, we obtained CD8+ T cells as being in early (E, naïve clusters), transitional (T, mostly CM and EM clusters) and late (L, EM and TE clusters) stages of differentiation (**Figures 2A, B**). Of the gene modules identified, those with distinct expression patterns across cell clusters after hierarchical clustering: modules 1, 3, 4, 7, and 9, along with module 8, 6, and 5, which encompassed more DEGs compared to modules they clustered with, were analyzed in detail and assigned with functions based on their most upregulated genes (**Figure 2B**, **Table 2**). To validate the ordering of cells at each stage of differentiation, we performed KEGG pathway analysis on differentially expressed genes among cell developmental stages and found that cells at E-stage are enriched for “Ribosome” pathway, at T-stage are enriched for multiple signaling pathways relevant to T cell activation, and at L-stage are associated with cytotoxic functions and pathological outcomes of viremia and of transplantation (**Figure 2C**, **Supplementary Figure S2B**). Similar properties were seen by GO term enrichment analysis (**Supplementary Figure S2C**). Five lineages with different ending nodes of each branch of the pseudotime trajectory, representing the transcriptional changes that CD8+ T cells with different fates undergo, were highlighted, revealing tracks that led to T-staged clusters T1: CM-c12 and T2: MAIT-c15, and L-staged clusters L1: TE-c21, L2: TE-c13 and L3: TE-c10 (**Figure 2D**). Modules 3, 7 and 4 each described the regional gene expression profiles at E-, T-, and L-stage cells, respectively (**Figure 2E**). Tracking cells along T-lineages, T1 ended in low cytotoxic T cell functions (Module 4) and T2 ended in high TCR signaling (Module 7) (**Figures 2E, F**). In L-lineages, as cells developed from early to later pseudotime, their Naïve and TSCM properties (Module 3) increased and then decreased, their cytotoxic T cell functions (Module 4) gradually increased and remained high but showed decline in L2 and L3 compared to L1 (**Figures 2E, F**). Modules 2, 5, 6, 1 and 9 had similar patterns in Lineages T1 and T2 but highlighted the differences between the three L-lineages in greater detail (**Figure 2E**). Overall, as cells differentiated, they experienced higher cell cycle regulation (Module 5 and 6) and lower cell division and proliferation (Module 1) and translational activities (Module 9), and these modules maintained a relatively low level of expression as cells continued differentiating (**Figures 2E, G**). As these lineages separated apart from the main trajectory around pseudotime of 30, L2 upregulated modules involved in cell cycle and proliferation regulation (Module 5 and 6) and downregulated modules involved in cell division (Module 1) and translation (Modules 9), reminiscent of senescent-like T cells, being terminally differentiated but with restricted proliferation and translational activity and high levels of cell cycle arrest (**Figures 2E, G**). Cells differentiating along L1 into TE-c21 likely represented circulating long-lived effector cells (LLECs) capable of sustaining a pool of potent effector that can rapidly respond to infections (**Figure 2G**). Compared to L1 and L2, cells following L3 to TE-c10 showed lower proliferative and terminal effector T cell function (Modules 1 and 2), likely representing non-CMV-associated populations (**Figure 2G**).

**Table 2:** Selected modules and assigned functions.

Module	Top 20 DEGs (ranked by Z-score)	Function
1	*B2M, S100A4, SH3BGRL3, TMSB4X, PFN1, ACTB, S100A6, CD52, GAPDH, TMSB10, CFL1, MYL6, LGALS1, IL32, CD99, HCST, CLIC1, ACTG1, CD3D, IFITM2*	Cell division and proliferation
2	*ZEB2, NEAT1, DDX5, PIK3R1, PTPRC, NR4A2, ATG2A, XIST, PPP2R5C, ADGRE5, RNF213, MYO1F, SRSF7, FUS, RUNX3, SYNE1, CEMIP2, ITGAL, KLF6, SYNE2*	Terminal differentiation
3	*LTB, IL7R, CCR7, MTRNR2L8, LEF1, SARAF, TRABD2A, PABPC1, FOXP1, RCAN3, TXK, NDFIP1, SATB1, TXNIP, VIM, MTRNR2L1, SERINC5, TCF7*	Naïve and stem-like memory T cell (TSCM) properties
4	*NKG7, CCL5, GZMH, CST7, GNLY, FGFBP2, GZMA, HLA-B, HLA-C, HLA-A, CTSW, IFIT2, IFIT3, EFHD2, FLNA, CYBA, KLRD1 HLA-DRB1, GZMB, GZMM*	Cytotoxic T cell functions
5	*DDX17, SMCHD1, PCSK7, NKTR, PPRC2C, CELF2, OGA, SFPQ, HNRNPU, MDM4, SRRM2, OGT, SMG1, ANKRD44, ARID1B, LENG8, RBM25, POLR2J3, SRSF11, CASP8*	Cell proliferation regulation
6	*MALAT1, ATM, LUC7L3, PNISR, AAK1, N4BP2L2, LIMD2, STK4, TNRC6B, SON, FYB1, TTC14, ZRANB2, PHF3, ATXN7, FTX, NAP1L4, SECISBP2, ETS1, RIPOR2*	Cell cycle regulation
7	*DUSP2, KLRB1, GZMK, UBC, JUNB, FTH1, SRGN, ZFP36L2, NFKBIA, ZFP36, CD74, JUND, DUSP1, CMC1, CXCR4, JUN, FOS, EIF1, ZNF331, SLC4A10*	TCR signaling
9	*TPT1, EEF1A1, EEF1B2, EEF1G, NACA, FAU, RACk1, GAS5, UBA52, NPM1, EIF3E, FXYD2, NOSIP, BTF3, LDHB, COX7C, TOMM7, MAL, CD27, PFND5*	Translational activity

### 3.3 CD8+ T cells during primary CMV infection adopted transcriptional programs with restrained proliferation and differentiation

**Figure 3:**
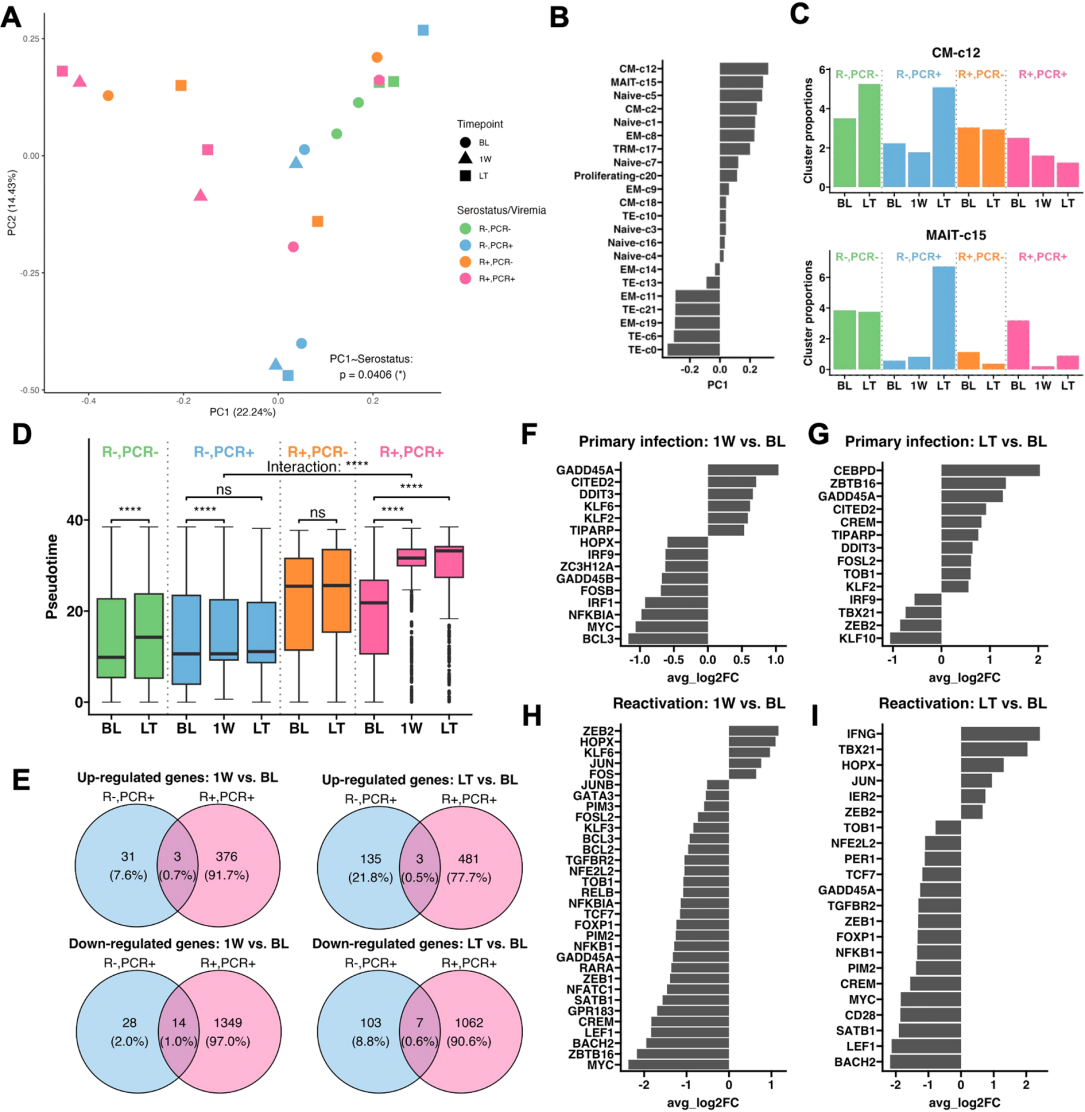
Transcriptional change in CD8+ T cells in CMV primary infection and reactivation. **(A)** PCA plot performed on cluster composition of each sample, colored by patient serostatus, viremia result, and timepoint. Mixed effect linear regression with patient identity as a random term was used to determine whether serostatus significantly affects each PC. **(B)** Loadings of clusters contributing to PC1. **(C)** Longitudinal cluster proportions of CM-c12 and MAIT-c15. **(D)** Box-and-whisker plots of cells at BL, 1W, and LT, separated by patient serostatus and viremia result. The lower and upper hinges of the boxes, lines within the boxes and the whiskers each represent the first and third quartiles, the mean, and the range of data no further than 1.5*inter-quartile range (IQR). Outliers are 1.5*IQR from the borders of the box and shown beyond the end of whiskers. Asterisks indicate p-values comparing the change in pseudotime of cells from BL to 1W and BL to LT in 4 groups of patients, and the interaction between patient serostatus and their change in pseudotime from BL to 1W (labeled as “Interaction”), determined by mixed effect linear regression, with patient identity as a random effect. Significance levels as defined in Methods. **(E)** Venn diagrams of unique and shared up- and down-regulated genes at 1W and LT over BL between primary infection and reactivation. Genes are differentially expressed if their adjusted p-value <0.05 and average Log2(Fold change (FC)) > 0.5 or <-0.5. **(F)** Average Log2(FC) of transcription factors and signal transducers that are differentially expressed at 1W over BL during primary infection, **(G)** at LT over BL during primary infection, **(H)** at 1W over BL during reactivation, and **(I)** at LT over BL during reactivation.

To further investigate CMV-driven CD8+ T cell fates, we performed principal component analysis (PCA) on cluster composition of each sample and found PC1 primarily distinguished samples by CMV serostatus (p =0.0406) (**Figure 3A**). Loadings of PC1 indicated that EM-c11, c19 and TE-c0, c6, c21 clusters strongly contributed to profiles of R+ patients, whereas CM-c12 and MAIT-c15 were the top 2 clusters associated with R- patients and the pseudotime lineages T1 and T2 (**Figure 3B**). Longitudinal expansion of CM-c12 in both PCR- and PCR+ R- patients indicated it’s likely non-CMV-related, but MAIT-c15 was expanded after primary CMV infection having been higher at BL in the PCR- group (**Figure 3C**). Elevated expression of genes involved in TCR signaling (Module 7) suggests they potentially have antiviral functions (**Figure 2F**).

Using pseudotime metric as an indicator of level of differentiation in each patient group, we found significantly advanced differentiation of CD8+ T cells in R+ PCR+ patients at 1W and remained high at LT (BL to 1W: p =7.98e-137; BL to LT: p =8.51e-138), whereas in R- PCR+ patients there was no significant LT variation (p =0.499) (**Figure 3D**). Additionally, while primary infection led to a significant 1W change in differentiation (BL to 1W: p =1.72e-08), it was significantly less pronounced than the 1W change after CMV reactivation (“Interaction”, p =1.22e-33) (**Figure 3D**). The lower magnitude of transcriptional changes in primary infection was also reflected in the longitudinal expressions of gene modules, where modules 3 and 9 had higher expression in R- compared to R+ patients, but their levels of expressions remained stable over time in R- patients (**Supplementary Figure S3**). We then asked whether specific genes were differentially expressed from BL to 1W and to LT during primary infection and reactivation (**Figure 3E**). Only 31 and 28 genes were up- and downregulated from BL to 1W in primary infection, whereas 376 and 1349 up- and downregulated genes were identified from BL to 1W in reactivation. At LT, though more DEGs were found, primary infection still had overall less transcriptomic change compared to reactivation. By analyzing transcription factors and signal transducers among the DEGs, we found that during 1W post-primary infection, transcripts involved in T cell activation [*ZC3H12A* (22), *NFKBIA* (23) and *BCL3* (24)] and downstream TCR signal transduction [*FOSB* (25), *MYC* (26), and *GADD45B* (27)] were downregulated, suggesting an impeded T cell response upon antigen-engagement (**Figure 3F**). Moreover, suppressors for T cell effector function and proliferation, *GADD45A* (28), *CITED2* (29), *DDIT3* (30), and *KLF6* (31) were upregulated (**Figure 3F**). Though *KLF2*, another upregulated transcription factor, has been reported to enhance effector T cell programing in CAR-T cells, it is also involved in restricting the migration of activated T cells (32, 33). At LT, an anti-proliferation program was also seen with upregulation of IL-2 suppressors [*CREM* (34) and *TOB1* (35)], and continued expression of suppressor genes upregulated at 1W (**Figure 3G**). In addition, transcription factors important for T cell renewal and differentiation, *TBX21* (T-bet) (36) and *ZEB2* (37, 38), were not upregulated (**Figure 3G**).

At 1W post-CMV reactivation, transcriptional programming of the CD8+ T cell memory response was accompanied by upregulated AP-1 family members *JUN/FOS* (39, 40) and *BCL2* (41), loss of naivety [downregulated *TCF7* (42, 43) and *LEF1* (42)], unimpeded cell proliferation (downregulated anti-proliferative and apoptotic genes *TOB1*, *GADD45A* and *CREM*), and enhanced terminal differentiation [upregulated *ZEB2* and *HOPX*, downregulated downstream effector transcriptional programs through *BCL3* and *BACH2* (44)] (**Figure 3H**). These trends were maintained at LT, coupled with downregulation of *CD28*, further substantiating memory inflation and terminal differentiation (**Figure 3I**).

### 3.4 CMV reactivation expands effector CD8+ T cell clusters essential for acute and long-term protection

**Figure 4:**
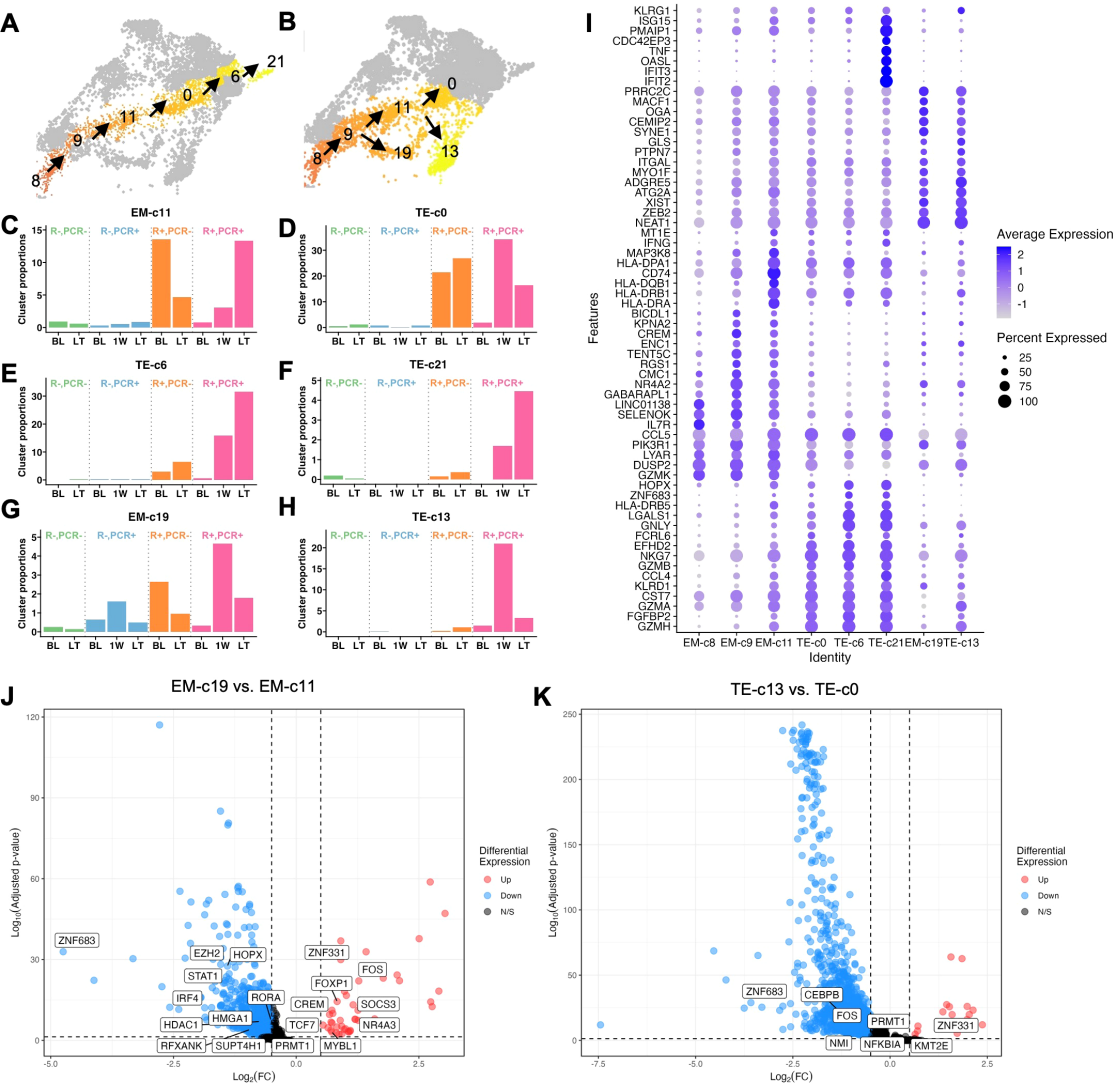
Longitudinal frequency and transcriptomes of CMV reactivation-expanded effector CD8+ T cell clusters. **(A)** Cluster localization in L1 and **(B)** L2. **(C-H)** Longitudinal frequencies of L1 and L2 clusters that explained variance of PC1 indicated in **Figure 3B**. **(I)** Dot plot of expression levels of KLRG1 and Top 10 markers of each cluster (p <0.05, average Log2(Fold Change) >0.5, difference in percentage of expression >0.1). **(J)** Volcano plot of DEGs comparing EM-c19 with EM-c11 and **(K)** TE-c13 with TE-c0. Transcription factors and regulators are labeled.

Clusters that accumulated in R+ patients resided in two lineages: L1, comprising a gradual progression of clusters EM-c11, TE-c0, TE-c6 and TE-c21 with increasing differentiation levels following EM-c8 and EM-c9, and L2, consisting of clusters EM-c19 and TE-c13 branching off from the main trajectory of EM-c8 and EM-c9 (**Figures 4A, B**). The four clusters in L1 exhibit a *KLRG1^hi^IL7R^lo^* phenotype, along with increasing expression of cytotoxic markers such as *GZMB*, *GNLY* and *IFNG* and transcription factors *ZEB2* and *ZNF683* (Hobit), suggesting a progressive differentiation into highly cytotoxic, terminally differentiated effector cells, likely specialized for immediate antiviral response (**Figure 4I**) (45). The less differentiated EM-c11 and TE-c0 clusters were observed at higher frequencies at BL in PCR- patients compared to PCR+ patients, suggesting they played a protective role against CMV reactivation (**Figures 4C, D**). The more differentiated TE-c6 and TE-c21 clusters were virtually absent in R- and PCR- R+ patients, but noticeably expanded longitudinally after reactivation (**Figures 4E, F**). TE-c0, positioned immediately upstream of TE-c6 in pseudotime, expanded acutely following reactivation but its frequency declined at LT (**Figure 4D**). Along with their effector functions, TE-c6 and TE-c21 likely represent cells differentiated from TE-c0 that remain elevated in number in the circulation to provide robust immune control in the long-term.

In L2, EM-c19 and TE-c13 were both induced acutely at 1W post-CMV reactivation (**Figures 4G, H**). Similarly high *ZEB2* expression suggested that these cells were committed to an effector differentiation pathway, with TE-c13 more differentiated than EM-c19, evidenced by higher *KLRG1* expression, but both clusters had less pronounced cytolytic activities as indicated by lower expression of effector molecule transcripts such as *IFNG*, *GNLY* and *GZMB* compared to L1 clusters (**Figure 4I**). To investigate the transcriptomic differences driving the distinct differentiation paths, we compared EM-c19 with EM-c11 and TE-c13 with TE-c0, as they appeared to differentiate in parallel, indicated by similar pseudotime values, and highlighted the transcription factors among DEGs (**Figures 4J, K**). While upregulation of *FOXP1, FOX* and *NR4A3* in EM-c19 suggested activation, functionality and proliferation were dampened, as evidenced by upregulation of *SOCS3 (* 46) and downregulated *PRMT1* (47), *STAT1* (48) and *IRF4* (49). This aligns with their senescent-like properties, based on their association with a gene module enriched for cell cycle regulation and longitudinal decline post-CMV reactivation (**Figures 2G**, **4J**). Fewer transcription factors were found differentially regulated between TE-c13 and TE-c0, but both EM-c19 and TE-c13 had a transcriptomic profile of *ZNF331* upregulation and *ZNF683* downregulation, suggesting effector-like nature of these cells, with attenuated inflammatory responses evidenced by downregulated *PRMT1*, *FOS* and *NFKBIA* (**Figure 4K**).

### 3.5 CD28^lo^ KLRG1^hi^ CD127^lo^ HLA-DR^hi^ CD8+ T cells associate with controlling CMV reactivation

**Figure 5:**
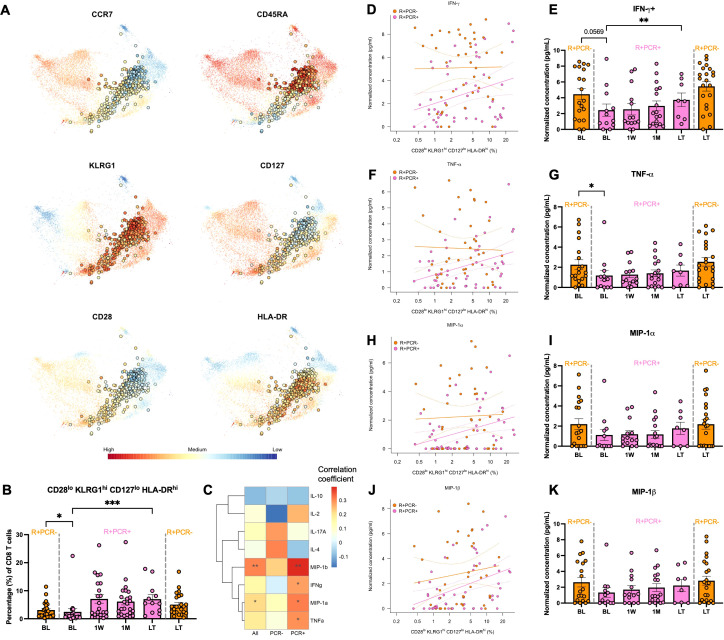
CD28^lo^ KLRG1^hi^ CD127^lo^ HLA-DR^hi^ cluster frequency and correlation with CMV control. **(A)** Flow cytometry was performed on PBMCs of 31 PCR+ and propensity score matched 31 PCR- KTRs to profile CD8+ T cells. Live CD3+CD8+ T cells were subject to unsupervised clustering (FlowSOM) and projected onto t-SNE. Expression levels of selected markers were highlighted for the cluster of CD28^lo^ KLRG1^hi^ CD127^lo^ HLA-DR^hi^ phenotypes. **(B)** Longitudinal frequencies of the CD28^lo^ KLRG1^hi^ CD127^lo^ HLA-DR^hi^ cell cluster of R+ PCR- patients (orange) at BL (n=24) and LT (n=24) and R+ PCR+ patients (pink) at BL (n=18), 1W (n=21), 1M (n=24) and LT (n=12). Error bars indicated standard error of mean (SEM). P-values comparing PCR- and PCR+ patients at BL and LT were determined by Mann Whitney test; Change over time after viremia in PCR+ patients was determined by mixed-effect linear regressions with patient identity as a random effect. **(C)** PBMCs were stimulated with CMV peptides or left unstimulated in 8 hours. Secretomes were analyzed using human 38-plex Luminex. Concentrations of analytes were correlated (Spearman) with proportions of CD28^lo^ KLRG1^hi^ CD127^lo^ HLA-DR^hi^ cell cluster in all R+ patients and PCR+/- patients separately. Correlation coefficients were colored in heatmap and p-values were annotated as asterisks. **(D)** Correlation of concentrations of CMV-stimulated IFN-γ secretion with CD28^lo^ KLRG1^hi^ CD127^lo^ HLA-DR^hi^ cell cluster frequency and their concentrations at longitudinal levels **(E)**. Correlations and longitudinal frequencies were depicted similarly for **(F, G)** TNF-α, **(H, I)** MIP-1α and **(J, K)** MIP-1β. Significance levels as defined in Methods.

Clusters EM-c11 and TE-c0 had higher proportions at BL in R+ patients who resisted CMV reactivation and subsequently expanded post-viremia in those who had reactivation, suggesting that cells of similar *KLRG1* ^hi^ *IL7R* ^lo^ phenotype may predict whether R+ patients would be susceptible to CMV reactivation post-Tx. We validated CD8+ T cell phenotypes in a cohort of 31 PCR+ KTRs and their propensity score-matched 31 PCR- KTRs by flow cytometry and identified a CD28^lo^ KLRG1^hi^ CD127^lo^ HLA-DR^hi^ cluster using FlowSOM unsupervised clustering (**Figure 5A**). Similar to single-cell clusters EM-c11 and TE-c0, flow cytometry-validated CD28^lo^ KLRG1^hi^ CD127^lo^ HLA-DR^hi^ cluster consisted of EM and TEMRA cells and was less frequent in R+ PCR+ patients at BL compared to R+ PCR- group (p =0.0163) and showed significant expansion over time post-reactivation (p =5.77E-04) (**Figures 5A, B**). To determine whether these cells may be functionally active in controlling CMV, we assessed CMV-induced cytokine and chemokine secretion by stimulating patient PBMCs with CMV peptides and correlated their concentrations with cluster proportion of CD28^lo^ KLRG1^hi^ CD127^lo^ HLA-DR^hi^ cluster (**Figure 5C**). CMV-stimulated effector cytokine IFN-γ (p =0.0494) and TNF-α (p =0.0358) secretion was strongly correlated with CD28^lo^ KLRG1^hi^ CD127^lo^ HLA-DR^hi^ cluster proportion in R+ PCR+ KTRs (**Figures 5C, D, F**). R+ PCR+ patients had suppressed production of IFN-γ in response to CMV at BL, yet the recall response recovered, increasing significantly from BL to LT (p =0.002) (**Figure 5E**). TNF-α production showed similar trends over time, with significantly lower capability at BL (p =0.0476) compared to PCR- group (**Figure 5G**). Chemokines MIP-1α (CCL3) and MIP-1β (CCL4) secretion also strongly correlated with CD28^lo^ KLRG1^hi^ CD127^lo^ HLA-DR^hi^ cluster proportion in PCR+ patients alone (MIP-1α: p =0.0262; MIP-1β: p=0.0043) and in all patients (MIP-1α: p =0.0416; MIP-1β: p=0.0031) (**Figures 5C, H, J**), and their longitudinal trends were like IFN-γ and TNF-α, although not statistically significant (**Figures 5I, K**).

## 4 Discussion

In this study, we delineated CD8+ T cell diversity and dynamics in response to CMV infection in KTRs by interrogating the transcriptional identities of CD8+ T cells at the single-cell level and determined the genes responsible for their positioning along the pseudotime trajectory from naïve to highly differentiated states. Our results showed that CD8+ T cell phenotypes form a continuum largely influenced by CMV exposure, both historical and current. Within this continuum we identified expansion of cells with MAIT cell signatures after CMV primary infection and cell trajectories leading to LLEC (L1) and senescent-like cell (L2) expansion after CMV reactivation. Along L1, *CD28^lo^ KLRG1^hi^ IL7R^lo^ HLA-DR^hi^* CD8+ T cells present in R+ patients prior to detection of viremia may act as a critical line of defense against CMV reactivation and serve as a source of LLECs for long-term viral surveillance in those who developed an infection, supported further by flow cytometry profiling of the complete cohort.

Our data revealed clear transcriptional differences of CD8+ T cell differentiation based on history of CMV infection. After primary infection, transcription factors that facilitate proliferation and effector-memory differentiation were not upregulated acutely or at long-term post-viremia. While one week might be too early for effector-memory differentiation to occur in naïve T cells, the absence of such signatures one year later still suggested obstructed T cell activation, providing transcriptional evidence of the lack in efficient generation and maintenance of CMV-specific memory response. Our data is consistent with other reports showing a lack or impaired cellular immunity to CMV in D+R- transplant recipients (50). In another study 30-40% of R- patients had CMV-responsive CD8+ T cells, yet the mean frequency of IFN-γ+CD137+CD8+ T cells was only 0.05% in R- compared to 0.64% in R+ patients (51). On the contrary, in a study of 11 R- patients, the authors reported CMV-specific T cell responses that were strong and comparable to R+ patients (52). While these data differ from ours and listed sources, it is possible that patient-to-patient variability in this small cohort could have influenced the results. The inability of R- patients to generate CD8+ T cell memory is likely due to a combination of CMV prophylaxis and maintenance immunosuppression. A recent study in D+R- liver transplant recipients compared those receiving preemptive antiviral therapy (PET) versus prophylactic antiviral therapy (PRO) and found lower proportions of polyfunctional CD8+ T cells in PRO group at 3, 6 and 12-months post-Tx (53). In addition, calcineurin inhibitors, a widely used component of immunosuppressive therapies, are more effective in preventing activation of naïve T cells than pre-existing memory T cells (54, 55). Interestingly, clusters with MAIT cell signatures at T-stage development with transcriptomic profiles similar to CM/EM clusters were found to be expanded LT post-primary infection. MAIT cells are considered as ‘innate-like’ due to their monomorphic MHC class I-related (MR1) molecule-restricted T cell receptor (TCR) and rapid response to bacterial and fungal antigens, and they can be activated independently of TCR through inflammatory cytokines IL-12, IL-15 and IL-18 (56–58). No functional role of MAIT cells has been established in CMV infection, but their frequency has been shown to be lower in CMV R+ healthy donors with high CMV antibodies compared to low-levels of antibodies, and in R+ HSCT recipients with high-level CMV reactivation compared to low-level reactivation (59–61). Although it is unknown whether MR1 can present CMV antigens, one study described immune evasion of CMV by downregulating MR1 *in vitro* (62). Given that MAIT cells may play a role in antiviral response, further evaluation of their abundance and functional properties in the context of CMV infection is required.

In R+ patients, we identified and validated CD28^lo^ KLRG1^hi^ CD127^lo^ HLA-DR^hi^ CD8+ T cells as a key feature distinguishing patients who did or did not develop detectable reactivation. These cells had higher frequencies at BL in PCR- than PCR+ groups and strong correlation with patients’ ability to produce IFN-γ and TNF-α in response to CMV stimulation, signatures of polyfunctionality of CD8+ T cells, and CCL3 and CCL4, chemoattractants that recruit activated CD8+ T cells (63, 64). In murine models, KLRG1^hi^CD127^lo^ cells have been termed as short-lived effector cells (SLECs), which are transient populations that achieve memory inflation by continuous replenishment (65). Later, this concept was challenged by the discovery of LLECs, which displayed a similar KLRG1^hi^CD127^lo^ phenotype and were capable of immediate cytotoxic effector functions, but remained long-lived in mice and human (66, 67). The cells we identified suggest that both theories – SLEC-driven memory inflation and LLEC-mediated long-term cytotoxicity – can coexist as a result of CMV infection, as our data support the concept of T cells existing along a continuum of differentiation. Within this continuum, KLRG1+ cells differentiate along the trajectory with increasing expression of terminal effector markers. Additionally, the evidence of TE-c0 long-term contraction and the pseudotime trajectory analysis suggest that this population can differentiate into later-stage TE-c6 and TE-c21, continuously replenishing them over time. The result is the continual expansion of TE-c6 and TE-c21, which were found at high frequencies one-year post-Tx. This dynamic supports a model where KLRG1+ progenitor cells not only provide immediate cytotoxic functions as SLECs but also contribute to the sustained pool of LLECs.

Consistent with features of long-lived effector CD8+ T cells induced by CMV described in the literature, cell clusters along L1 exhibit potent cytolytic potential with increasing transcription factors *ZEB2* and *ZNF683* (Hobit), both of which are markers of terminal differentiation (68). Studies in mice have defined ZEB2 as a transcriptional repressor that drives CD8+ T cells towards a fully functional, terminally differentiated cytotoxic state while limiting the generation of memory subsets (37, 38). Hobit, a homolog of Blimp-1, has been reported to express in quiescent state CMV-specific effector CD8+ T cells capable of immediate IFN-γ production in humans (69). Together, CD8+ T cells persist at terminal stage of effector differentiation for an extended period even in the absence of clinically detectable CMV. In contrast, acute 1W expansion followed by long-term contraction of L2 clusters EM-c19 and TE-c13 suggests different transcriptomic programs. With high expression of genes associated with cell cycle arrest and defective killing abilities reflected by higher *GZMK* profile versus *GZMB* and *GNLY*, these cells aligned with characteristics of cellular senescence (reviewed in (67)). In addition, these cells have relatively high *ZNF331* and low *ZNF683* expression compared to cell clusters with similar levels of differentiation. One study reported *ZNF331* expression in T cell clusters in breast cancer with resident effector memory phenotype co-expressing regulatory elements that may involve in cell cycle regulation and upregulating *GZMK* over *GZMB* and *PRF1*, aligning with our observations (70). With limited information on *ZFN331*, it remains unclear whether these cells expand in an antigen-dependent manner, to what extent they can contribute to antiviral immunity and whether their limited cytotoxic activity is a result of immunosuppression. Identifying the interaction partners of *ZNF331* and *ZNF683* in T cells and exploring the genes regulated by these transcription factors will enhance our understanding of the heterogeneity of CMV-induced CD8+ T memory recall response of R+ patients.

Current strategies to mitigate late-onset CMV infection or disease include risk stratification based on D/R CMV serology mismatch, clinical monitoring of DNAemia and/or assessment of absolute lymphocyte count or CMV-specific cell-mediated immunity (CMI) pre-transplant or at the termination of CMV prophylaxis (71–74). The presence of CD28^lo^ KLRG1^hi^ CD127^lo^ HLA-DR^hi^ CD8+ T cells prior to detection of viremia may serve as additional key biomarker for predicting which patients are likely to resist or develop CMV infection, potentially guiding early care and treatment strategies. The observations that they are not end-stage effectors, but instead retain the potential to establish long-lived capabilities, make them an ideal model for designing adoptive transfer strategies using engineered cells in patients that are vulnerable to refractory CMV infection. However, it is important to note that these cells are generated by prior CMV exposure in R+ patients, limiting the applicability of this approach in R- patients.

This study highlighted potential new and important findings related to CMV-mediated CD8+ T cell differentiation in KTRs and practicality of using immunophenotyping to predict CMV viremia, but limitations remain. First, although the single-cell experiments were performed on propensity matched KTRs to control for patient heterogeneity and we validated cell phenotypes identified in the single-cell experiment using flow cytometry in the larger, complete cohort, expanding the number of participants in future studies will be beneficial. In addition, while our KTR cohort all followed standard immunosuppressive regimen, the duration of prophylaxis varied based on CMV serostatus and could have affected the CD8+ T cell response observed. Despite smaller sample size, longitudinal trends of immune profiles and functionality and their associated transcriptomic signatures align with other studies in transplant (4, 15, 51, 53, 60, 68). Second, the measure of the cytokine secretome of PBMCs responding to CMV peptide stimulation may be confounded by immune cells other than CD8+ T cells. To overcome it, CMV peptide pools were designed to encompass the most immunodominant T cell antigens and exclude those that activate NK receptors. Additionally, we used a relatively short stimulation (8 hours), to preferentially assess CD8+ T cell memory response. Third, while our study primarily focuses on the effects of CMV – the most common virus encountered by transplant recipients – on CD8+ T cells, it is important to acknowledge that other viruses, such as Epstein-Barr virus (EBV), herpes simplex virus (HSV), and BK polyomavirus could also drive differences in CD8+ T cells. We recognize this as a limitation and associated the key CD28^lo^ KLRG1^hi^ CD127^lo^ HLA-DR^hi^ CD8+ T cell population with measurements of CMV-induced cellular immunity to underscore the unique impact of CMV. Whether similar CD8+ T cell phenotypes could be observed in response to other viruses remains an open question that warrants investigation.

In conclusion, CMV infection induces a continuum of transcriptionally diverse CD8+ T memory cells across time. Notably, persistence of immunity to CMV in R- KTRs experiencing primary infection was significantly impaired, and in R+ KTRs CMV reactivation control was associated with increased frequency of CD28^lo^ KLRG1^hi^ CD127^lo^ HLA-DR^hi^ CD8+ T cells, which have the potential to differentiate into long-lived effector cells to provide durable immunosurveillance. Our data provided transcriptional insights into CD8+ T cell differentiation along different trajectories and underscore the possibility of using cellular markers to predict viremia onset.

## Data Availability

The datasets presented in this study can be found in online repositories. The names of the repository/repositories and accession number(s) can be found below: https://www.ncbi.nlm.nih.gov/bioproject/PRJNA745955, PRJNA745955 https://immport.org/shared/study/SDY1600, SDY1600.
