# Supplementary material for: Cytotoxic KLRG1+ IL-7R- effector CD8+ T cells distinguish kidney transplant recipients controlling cytomegalovirus reactivation
Source: Front Immunol. 2025 Feb 14;16:1542531. doi: 10.3389/fimmu.2025.1542531 (PMC11868092; doi:10.3389/fimmu.2025.1542531)
Supplement: Supplementary file 1 [file DataSheet1.docx]

Supplemental Materials and Methods for

**Cytotoxic KLRG1+ IL-7R- effector CD8+ T cells distinguish kidney transplant recipients controlling Cytomegalovirus reactivation**

Yumeng Sun^1^, Subha Sen^1^, Rajesh Parmar^1^, Janice Arakawa-Hoyt^2^, Monica Cappelletti^1^, Maura Rossetti^1^, David W. Gjertson^1^, Tara K. Sigdel^5^, Minnie M. Sarwal^5^, Joanna M. Schaenman^3^, Suphamai Bunnapradist^4^, Lewis L. Lanier^2^, Harry Pickering^1#^, Elaine F. Reed^1#^* and the CMV Systems Immunobiology Group^6^

^1^Department of Pathology and Laboratory Medicine, University of California, Los Angeles, Los Angeles, California, USA

^2^Department of Microbiology and Immunology, Parker Institute for Cancer Immunotherapy, University of California, San Francisco, California, USA

^3^Division of Infectious Diseases, David Geffen School of Medicine, University of California, Los Angeles, Los Angeles, California, USA

^4^Division of Nephrology, David Geffen School of Medicine, University of California, Los Angeles, Los Angeles, California, USA

^5^Department of Surgery, Division of Multi Organ Transplantation, University of California, San Francisco, San Francisco, CA, USA

^6^CMV Systems Immunobiology Group (in alphabetical order): Victoria Adebambo, Janice Arakawa-Hoyt, Patrick Boada, Jenny Brook, Suphamai Bunnapradist, Jim Cimino, Izabella Damm, Nakul Datta, Mario Deng, Don Diamond, Tin Doung, David Elashoff, Janette Gadzhyan, David Gjertson, Alexander Hoffman, Kenichi Ishiyama, Maggie Kerwin, Lewis Lanier, Megan Llamas, Erik Lum, Dane Munar, Rajesh Parmar, Claudia Perez, Harry Pickering, Priyanka Rashmi, Elaine F. Reed, Maura Rossetti, Dmitry Rychkov, Minnie Sarwal, Joanna Schaenman, Subha Sen, Tara Sigdel, Danielle Sim, Marina Sirota, Jun Shoji, Yumeng Sun, Swastika Sur, Parhom Towfighi, Flavio Vicenti, Otto Yang.

^#^Authors contributed equally to this work

*Corresponding Author: Elaine F. Reed, Ph.D., Department of Pathology and Laboratory Medicine, University of California, Los Angeles, Los Angeles, California, USA, [ereed@mednet.ucla.edu](mailto:ereed@mednet.ucla.edu), +1 (310) 794-4943

**Supplemental Methods**

**Patient enrollment and study design and clinical management**

All patients provided informed consent to participate in the research in full adherence to the Declaration of Helsinki. The clinical and research activities being reported are consistent with the Principles of the Declaration of Istanbul as outlined in the Declaration of Istanbul on Organ Trafficking and Transplant Tourism. Consented kidney transplant recipients were enrolled after transplantation at Ronald Reagan Medical Center of University of California, Los Angeles (IRB#11-001387). Written informed consent was received prior to participation. Age and sex-matched KTRs (31 CMV R+ and 31 CMV R-) transplanted with a CMV-seropositive donor (D+) were selected for study and their clinical characteristics previously described. Blood was drawn from these patients before the detection of CMV viremia (Baseline, BL, approximately 3 months post-transplant), 1-week post-viremia (1W), 1-month post-viremia (1M) and 12 months post-transplant (Long-term, LT), and their peripheral blood mononuclear cells (PBMCs) were isolated and cryopreserved. In addition, CMV PCR- KTRs were selected as controls after matching them with PCR+ groups using nearest-neighbor matching based on propensity scores derived from a logit model, considering recipient age, sex, race, induction therapy, and CMV serostatus before transplant. Patients received induction therapy with either anti-thymocyte globulin (ATG) or basiliximab and were maintained on triple immunosuppression, including tacrolimus, mycophenolate mofetil, and prednisone. CMV viremia (CMV PCR+) was defined as the presence of CMV DNA exceeding 137 IU/mL in the patient's blood through PCR test (Cobas AmpliPrep/Cobas TaqMan CMV test, Roche).

**Single-cell RNA sequencing**

Four CMV PCR+ patients (2 R- and 2 R+) along with their matched PCR- control patients (2 R- and 2 R+), were chosen for the study. Individual patient samples consisting of 4,000 cells each were processed using Chromium Single Cell 3' kits from 10X Genomics. These samples were then sequenced using the NovaSeq S2 instrument. The raw fastq files, containing gene expression data, were analyzed using Cell Ranger 5.0.1 to create a gene expression count matrix. Subsequently, data from all patients were integrated using the Cell Ranger 'aggr' function. A total of 36,001 genes in 87,493 cells were sequenced.

**Single-cell transcriptomic data analysis**

Cells exhibiting fewer than 250 unique features (genes), 500 total RNA counts, Log_10_(Genes per Unique Molecular Identifier (UMI)) less than 0.8, or a mitochondrial count exceeding 15%, were excluded. Genes expressed in less than 10 cells were also excluded. Data acquired from experiments in different batches were integrated using *SelectIntegrationFeatures*, *FindIntegrationAnchors*, and *IntegrateData* functions to correct for batch-effect. Cells were clustered using *FindNeighbors* (dims =1:50) and *FindClusters* functions (resolution =2) using their total gene expressions and visualized using t-distributed stochastic neighboring embedding (t-SNE) through *RunTSNE* function. Distinct non-CD8+ T clusters that separated from CD8+ T cell clusters were validated as B cells (*MS4A1+),* CD4+ T cells (*CD4+),* NK cells (*CD3D-NCAM1+KLRF1+*)*,* γδ T cells (*TRAC-CD8A/B-TRDC+TRGC1/2+*), and platelets (*PPBP+*) and were removed prior to further downstream analyses. To aid visualization, cells of each sample were subsampled to 1,000 cells (if more than 1,000 cells present). Subsampled non-normalized data of each sample were re-integrated and subject to normalization process per Seurat’s vignette. Briefly, we used *ScaleData*, *RunPCA* (3,000 most variable genes, 50 principal components), *FindNeighbors*, and *FindClusters* functions. 22 distinct clusters of CD8+ T cells were identified at resolution= 1.4 and UMAP was generated at n.neighbors =10 and min.dist =0.05 using *RunUMAP* function. UMAP projections of cells colored by patient identity parameters and genes were produced using *DimPlot* and *FeaturePlot* functions, respectively. Differentially expressed genes (DEGs) were identified using *FindMarkers* to compare two groups or *FindAllMarkers* functions to compare one group versus the rest of the cell groups. Significant genes were selected based on statistical threshold of Log_2_(Fold Change) >0.5 and two-sided adjusted p-value <0.05. Gene set enrichment of DEGs was performed using *compareCluster* function and plotted with *dotplot* function from clusterProfiler package (Version 4.6.2)^1,2^. The Gene Ontology (GO) and Kyoto Encyclopedia of Genes and Genomes (KEGG) databases were used for pathway annotation.

**Inference of pseudotime developmental trajectories for CD8+ T cells.**

The Seurat object was converted to CellDataSet object using *as.cell_data_set* function and was subject to single-cell trajectory construction according to Monocle3’s vignette. Pseudotime trajectories were constructed using *learn_graph* (partition =F, close_loop =F). Root of the trajectory was specified using *get_earliest_principal_node*, a helper function from developer which identifies the root, to select the node that is most occupied by cells at baseline. Cells were then ordered in pseudotime using *order_cells* via the root_pr_node =get_earliest_principle_node argument. Genes that vary across cells in the UMAP space were identified using *graph_test* function. DEGs (q_value <0.05) were then subject to grouping using *find_gene_modules* at resolution = 0.001, and *aggregate_gene_expression* function to calculate module expression of each cluster or each cell. Lineages were defined using *choose_graph_segments* function, in which the root was chosen as the ‘starting node’ and the last nodes on the branches of the trajectory were chosen as ‘ending nodes.’ Pseudotime values of each cell were extracted using *pseudotime* function.

**References**

1. Wu T, Hu E, Xu S, et al. clusterProfiler 4.0: A universal enrichment tool for interpreting omics data. The Innovation. 2021;2(3):100141. doi:10.1016/j.xinn.2021.100141

2. Yu G, Wang LG, Han Y, He QY. clusterProfiler: an R Package for Comparing Biological Themes Among Gene Clusters. OMICS. 2012;16(5):284-287. doi:10.1089/omi.2011.0118

**Supplemental Tables**

**Supplemental Table 1.** **T cell antibody panel for flow cytometry**

| Fluorochrome | Marker | Clone |
| --- | --- | --- |
| BV421 | CD25 | BC96 |
| BV510 | CCR4* CRTH2 | BM16 |
| BV605 | CXCR3 | G025H7 |
| BV650 | HLA-DR | L243 |
| BV711 | CD28 | CD28.2 |
| BV785 | CD45RA | HI100 |
| FITC | CD57 | HNK-1 |
| PE | KLRG1 | SA231A2 |
| PE-CY7 | PD-1 | EH12.2H7 |
| PE-CF594 | CD127 | A019D5 |
| PCP-CY5.5 | CD4 | A161A1 |
| APC | CCR7 | G043H7 |
| A700 | CD8 | SK1 |
| APC/Cy7 | CD3 | HIT3a |
| BUV395 | CXCR5 | RF8B2 |
| BUV496 | Viability | Invitrogen™ LIVE/DEAD™ Fixable Blue Dead Cell Stain Kit |
| BUV737 | CCR6 | 11A9 |

**Supplemental Figures**


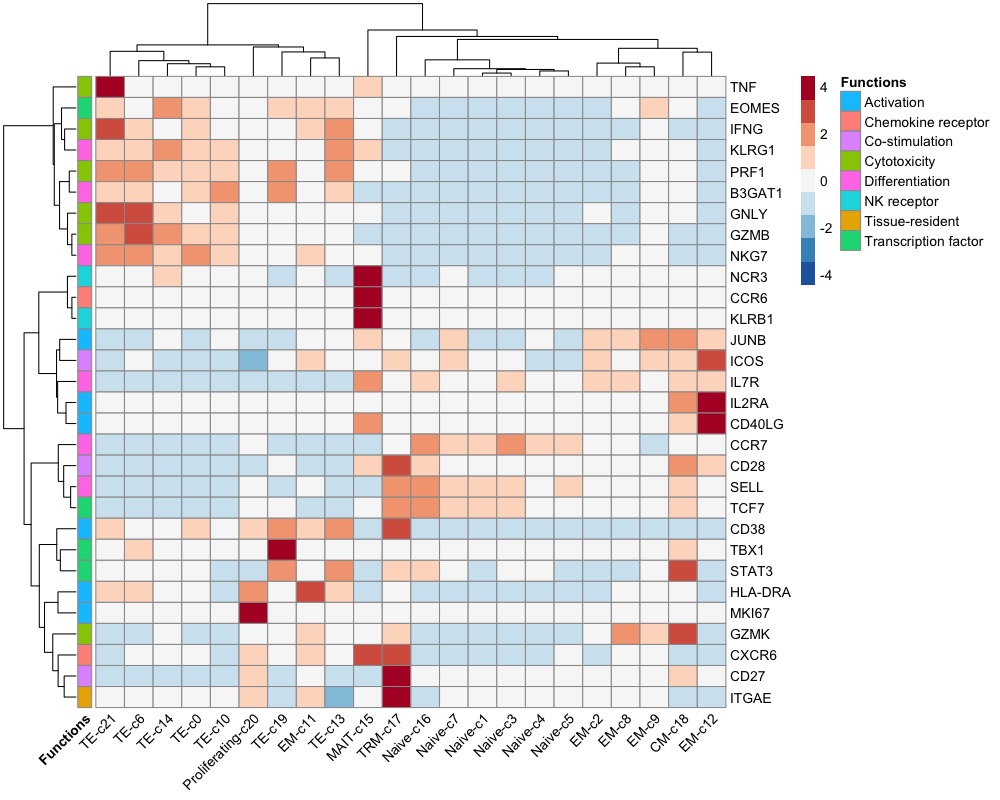


**Supplemental Figure 1**. Heatmap of selected canonical markers of CD8+ T cell subsets and their expression in all clusters.


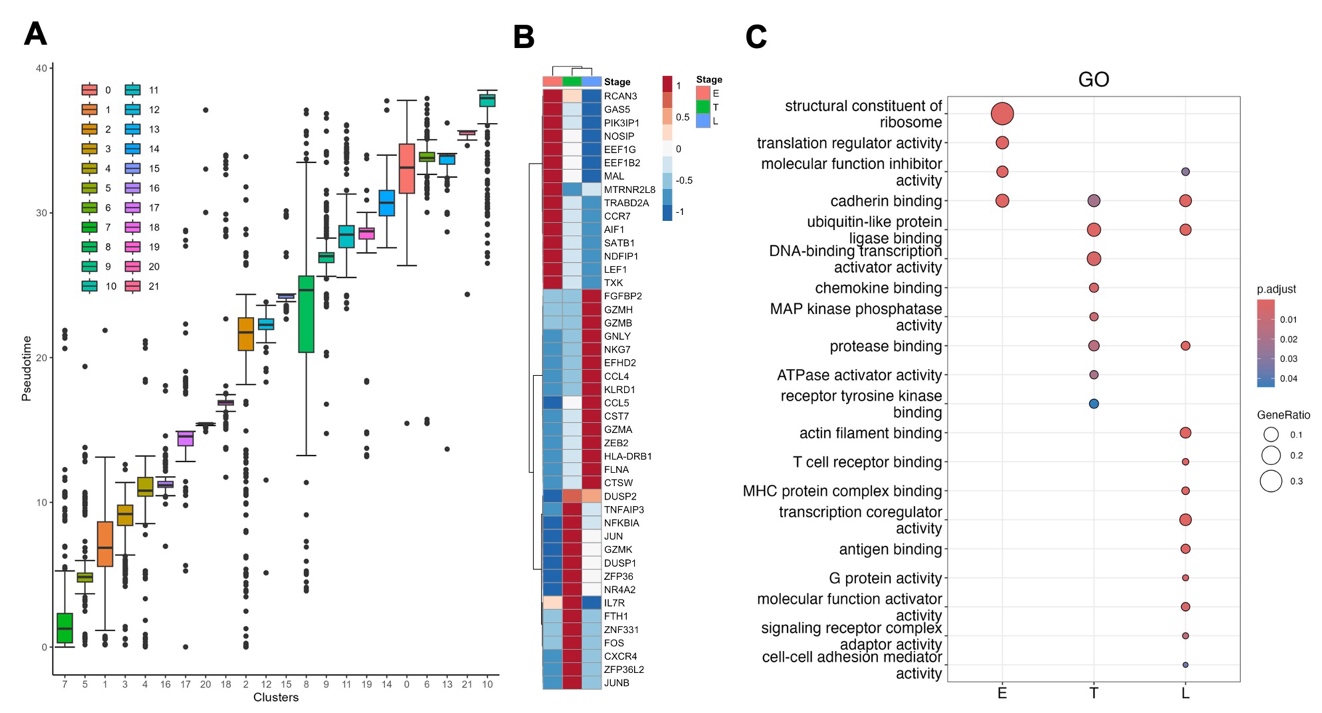


**Supplemental Figure 2. CD8+ T cell differentiation and assigned pseudotimes.** (**A**) Pseudotime values of cells in each cluster, ranged from earliest to latest pseudotime. (**B**) Top 15 DEGs comparing E, T, and L-staged cells defined in **Figure 2**, and (**C**) their enriched GO terms.


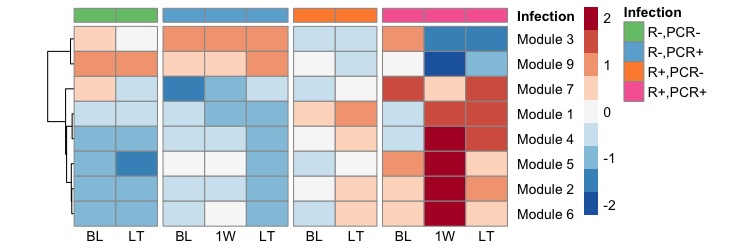


**Supplemental Figure 3.** Expression of selected gene modules at BL, 1W and LT of CD8+ T cells from patients with different infection group.
